# Research progress on the anti-tumor effect of Naringin

**DOI:** 10.3389/fphar.2023.1217001

**Published:** 2023-08-17

**Authors:** Jing He, Hui-Ping Zhang

**Affiliations:** ^1^ First Clinical Medical College, Shandong University of Traditional Chinese Medicine, Jinan, China; ^2^ Oncology Department, Jinan Traditional Chinese Medicine Hospital, Jinan, China

**Keywords:** Naringin, anti-tumor, mechanism of action, research progress, cancer

## Abstract

Naringin is a kind of natural dihydro flavone, which mainly exists in citrus fruits of the Rutaceae family, as well as traditional Chinese medicines such as trifoliate orange, fingered citron, exocarpium citri grandis, and rhizoma dynamite. Modern pharmacological studies have shown that Naringin has excellent anti-tumor activity. Through reviewing the relevant literature at home and abroad in recent years, we summarized the pharmacological mechanism of Naringin to play an anti-cancer role in blocking tumor cell cycle, inhibiting tumor cell proliferation, inducing tumor cell apoptosis, inhibiting tumor cell invasion and metastasis, inducing tumor cell autophagy, reversing tumor cell drug resistance and enhancing chemotherapeutic drug sensitivity, as well as anti-inflammatory to prevent canceration, alleviate Adverse drug reaction of chemotherapy, activate and strengthen immunity, It provides theoretical basis and reference basis for further exploring the anticancer potential of Naringin and its further development and utilization.

## 1 Introduction

**FIGURE 1:**
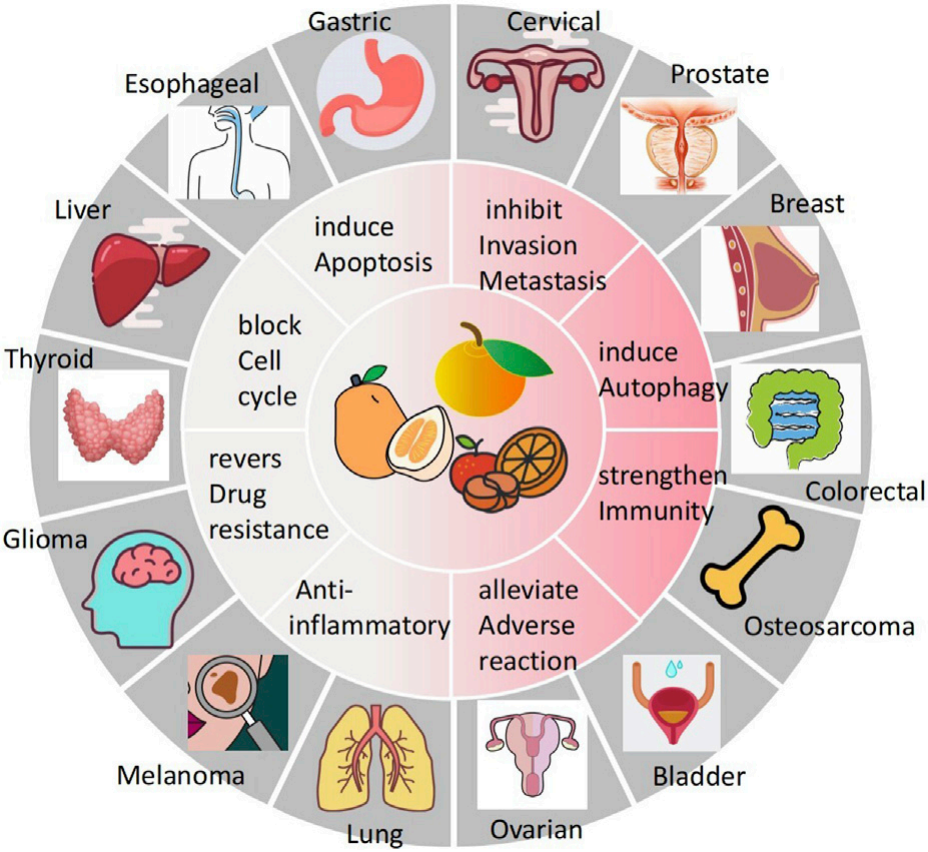
Naringin has broad-spectrum anticancer pharmacological activity, which can inhibit the occurrence and development of cervical cancer, gastric cancer, prostate cancer, breast cancer, colorectal cancer, Osteosarcoma, bladder cancer cancer, ovarian cancer, Melanoma, Glioma, lung cancer, Esophageal cancer cancer, thyroid cancer, and liver cancer.

**FIGURE 2:**
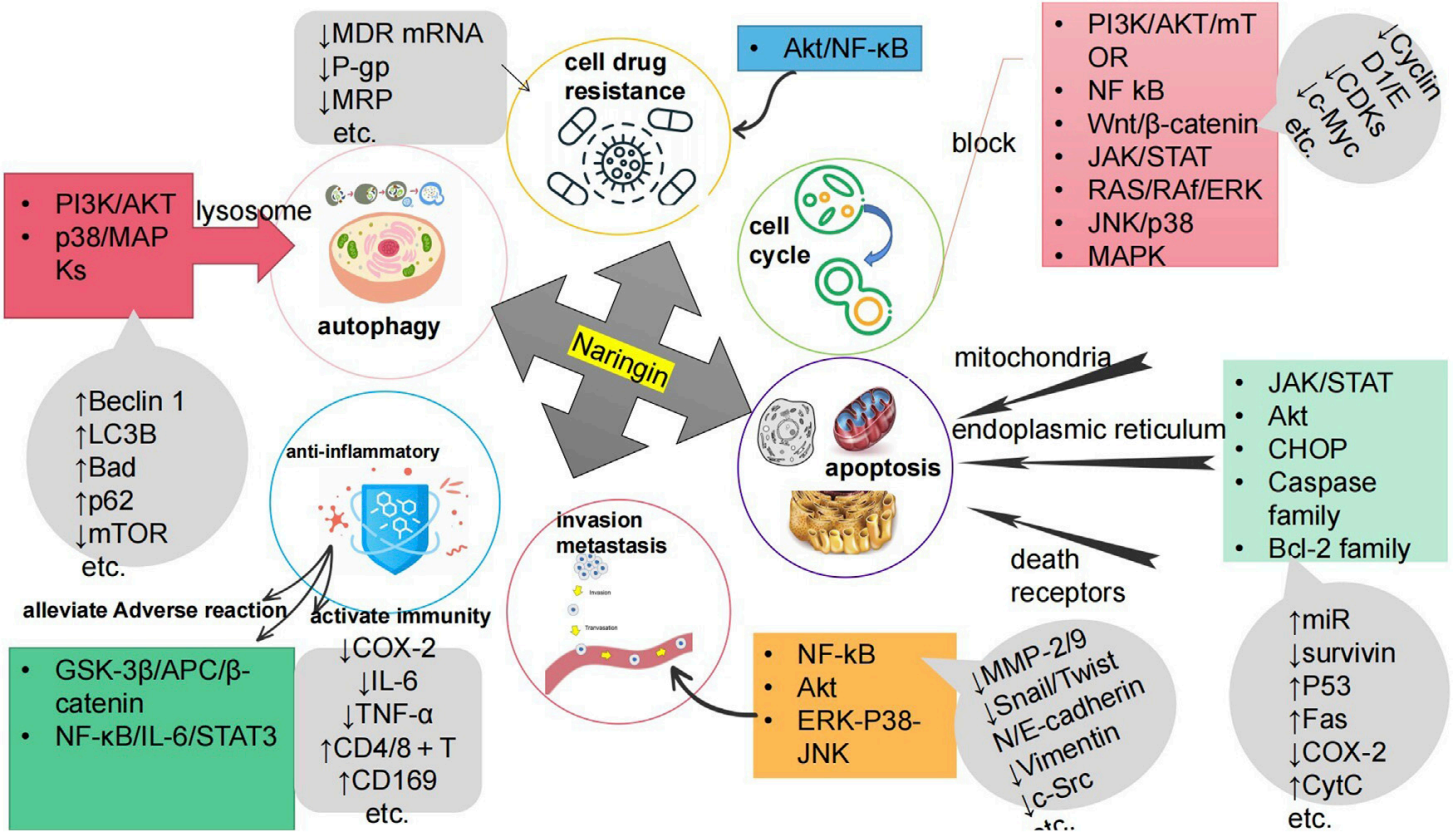
The anti-cancer mechanism of Naringin is to inhibit the proliferation of tumor cells by blocking the tumor cell cycle; Inducing tumor cell apoptosis; Inhibiting tumor cell invasion and metastasis; Inducing autophagy of tumor cells; Reversing tumor cell resistance and enhancing chemotherapy drug sensitivity; Anti inflammation to prevent cancer; It can reduce Adverse drug reaction of chemotherapy, activate and strengthen immunity.

Cancer poses a great threat to human health and is currently a major global public health problem and a major cause of disease burden in society. Natural products derived from plants and animals have a wide range of pharmacological activities (Xiaokaiti and Li, 2020), among which flavonoids distributed in the plant kingdom have significant anticancer properties (Nath et al., 2023). Naringin (Nar) is a natural dihydro flavonoid, mainly found in the skin and flesh of Rutaceae fruits, such as Grapefruit and citrusthe and herbal medicines such as Fructus Aurantii, Citrus aurantium, Bergamot, Citri Grandis Exocarpium, Drynariae Rhizoma (Yuan-bao et al., 2018), in addition to antioxidant, anti-inflammatory, hypoglycemic, lipid-lowering, cardiovascular protection, neuroprotection, gastrointestinal protection, prevention of osteoporosis and bone damage (Bharti et al., 2014; Chen et al., 2016), recent domestic and foreign studies have found that also has anti- Cervical Cancer, Gastric Cancer, Prostate Cancer, Breast Cancer, Colorectal Cancer, Osteosarcoma, Bladder Cancer, Ovarian Cancer, Melanoma, Glioma, Lung Cancer, Esophageal Cancer, Thyroid Cancer, Liver Cancer, and many other effects (Figure 1). The anti-tumor mechanism of Naringin is mainly to block tumor cell cycle, inhibit tumor cell proliferation, induce tumor cell apoptosis, inhibit tumor cell invasion and metastasis, regulate autophagy, reverse drug resistance and enhance the sensitivity of chemotherapy drugs, as well as anti-inflammatory, reduce the adverse reaction of chemotherapy Adverse drug reaction, activate and strengthen immunity, etc. (Figure 2) in a multi-target, multi-pathway and multi-level way to deter tumorigenesis and progression by regulating the relevant dysregulated signaling cascade responses (Xin-rong et al., 2022). It can be seen that Naringin is a drug candidate with great potential for tumor prevention and treatment, and has broad prospects for development and utilization. At present, there is a lack of generalization and analysis of the molecular targets and pathways related to the regulation of Naringin in various tumors. Therefore, this paper reviews the literature on the anti-cancer mechanism of Naringin in recent years, aiming to provide some reference for its subsequent development and clinical application.

## 2 Blocking tumor cell cycle and inhibiting tumor cell proliferation

(The cell cycle refers to the basic life activity process of a cell from the end of the last division to the end of the next division, which is closely regulated and occurs in an orderly manner. When the regulation is disrupted, normal cells will experience uncontrolled growth and can transform into tumor cells (Liu et al., 2021). The proliferation of cancer cells is closely related to abnormal cell cycles, and by interfering and blocking the cell cycle, it can inhibit the continued division and proliferation of tumor cells (Jamasbi et al., 2022). Signal pathways are important mechanisms for information transmission within and between cells, controlling various physiological and pathological processes of cells (Mohan et al., 2022). A large number of studies have shown that Naringin can inhibit the proliferation of various tumor cells and tumor growth in a concentration time dependent manner. Exploring how Naringin targets to block cell cycle nodes under the mediation of multiple signal pathways and related molecules to inhibit tumor cell growth and proliferation is the primary mechanism to reveal its anti-cancer effect.

### 2.1 Blocking the cell cycle at various stages

The cell cycle is the basic process of cell proliferation, which includes four stages: G1, S, G2, and M. Cyclin D1/E (Qie and Diehl, 2016) is the regulator of cell Cyclin-dependent kinase CDKs (CDK2, CDK4, CDK6, etc.). The transcription product C-myc (Kim et al., 2015) encoded by oncogenes, as well as cell cycle inhibitory proteins p21, p53, p-Rb, etc., are important cell cycle participating factors.

In the study of gastric cancer SNU-1 (Xu et al., 2021) cells and Cervical cancer C33A, SiHA, HeLa cells (Lin et al., 2020), Naringin blocked PI3K/AKT pathway and β- Catenin/GSK-3 β Pathways to reduce the expression of cell cycle survival proteins c-Myc, CDK2, CDK4, and Cyclin D1, and increase the expression of p21/cip1 and p27/kip1. According to Erdogan et al. (Erdogan et al., 2018), in addition to p21 and p27, the expression of p53 in prostate cancer PCa cells treated with Naringin was also significantly increased, while the expression of NF kB p53 protein was significantly decreased; Li et al. (Li et al., 2013) found that Naringin can regulate Wnt in MDAMB-231 and BT-549 cells of triple negative breast cancer/β- The catenin pathway upregulates the expression of p21 protein and downregulates the expression of p-Rb, Cyclin D1, and Cyclin E. The results of the above four studies all indicate that Naringin can induce cell cycle arrest in G0/G1 phase. It is reported that (Liqiong and Na, 2020), Naringin inhibits cyclinD1 in colon cancer SW620 cells by up regulating the expression of tumor suppressor gene ARHI and p21 protein; Naringin inhibits the expression of Cyclin D1 in Osteosarcoma cells MG63 and U2OS by down regulating the transcription inhibitor Zeb1 (Ming et al., 2018), which blocks the G1 phase cell cycle to a certain extent, thus improving the inhibition rate of tumor cell proliferation (Yi et al., 2018). Kim et al. (Kim et al., 2008) found that Naringin can increase the expression of p21WAF1/cip1, downregulate cyclin D1/E, CDK2, CDK4, and induce G1 phase arrest in bladder cancer 5637 cells, which may be related to the inhibition of Ras/Raf/ERK signaling pathway. In another observation on the combined use of ovarian cancer SKOV3/CDDP cells (Zhu et al., 2020), Naringin combined with cisplatin downregulated Wnt/catenin signaling pathway β- The expression of catenin, c-Myc, and cyclin D1 prevents cell cycle progression from G1 phase to S phase. Ramesh et al. identified cervical cancer SiHA cells (Ramesh and Alshatwi, 2013). Exposure to the growth inhibitory concentration of Naringin resulted in a significant increase in the proportion of cells in G2/M phase and a decrease in cells in G1/S phase, suggesting that Naringin induced cell cycle arrest in G2/M phase.

### 2.2 Multiple signaling pathways involved in inhibiting tumor cell proliferation

Cheng (Cheng et al., 2020a) and Raha (Raha et al., 2020) found that in colorectal cancer SW620, HCT116 cells and gastric cancer AGS cells, Naringin inhibits the proliferation of malignant tumor cells by inhibiting PI3K/Akt/mTOR signaling pathway and down regulating the phosphorylation level of mammalian Sirolimus target (mTOR); Similarly, in non-small cell lung cancer A549 and H460 cells (Zhongyuan et al., 2017), Naringin inhibits the proliferation and growth of NSCLC cancer lines by downregulating the expression of the downstream Signaling molecule p70S6K of mTOR1. Bing-yu et al.'s research on Melanoma A375 cells (Bing-yu et al., 2016) and glioma U87 (Wei et al., 2020) cells shows that Naringin can reduce the level of mitotic protein kinase CDK4/CDK6 to inhibit its proliferation; Ruiying et al. (Rui-ying et al., 2022) found that Naringin inhibits the proliferation of cervical cancer ME-180 cells by increasing the expression of miR-628-5p; Zhou et al. (Zhou et al., 2019) showed that Naringin can also play a role in inhibiting the growth of Thyroid neoplasm. The above three studies have found that the anti-tumor effect of Naringin is related to its inhibition of PI3K/AKT pathway. Chen et al. (Chen et al., 2018) treated non small cell lung cancer H69 cells with different concentrations of Naringin for 24 h, the results showed that Naringin could reduce the phosphorylation level of vascular cell adhesion molecule 1 (VCAM-1 protein) and activate miR-126, which is related to the regulation of NF in H69 cells- κ The B signal pathway is related. Guo et al. (Guo et al., 2016) found that the production of lactic acid and ATP in Melanoma A375 and A875 cells significantly decreased after the action of Naringin, suggesting that Naringin can inhibit aerobic Glycolysis and play an anti proliferative activity. Further research shows that this is due to Naringin inhibiting the phosphorylation of non Receptor tyrosine kinase (c-Src), M2 type Pyruvate kinase (PKM2), Lactate dehydrogenase A (LDHA) and Hypoxia-inducible factor (HIF-1 α) The expression of glucose is related to the inhibition of glucose metabolism in A375 and A875 cells. About 80% of breast cancer tissues highly express estrogen receptor (ER), and Naringin plays an anti estrogen and anti aromatase activity role by regulating estrogen signal transduction and aromatase inhibition (El-Kersh et al., 2021), thereby inhibiting the proliferation of breast cancer cells. Feng et al. (Feng et al., 2021) observed a significant decrease in p-STAT3/STAT3 and p-JAK2/JAK2 ratio after Naringin was applied to esophageal cancer Eca109 cells, suggesting that Naringin blocked the activation of protein Tyrosine kinase signal transducer/activator of transcription (JAK/STAT) signal pathway, thereby inhibiting the proliferation and colony formation of esophageal cancer cells.

To sum up, Naringin mainly inhibits PI3K/AKT/mTOR, NF kB, Wnt/β-catenin signaling pathways such as catenin, JAK/STAT, RAS/RAf/ERK, JNK/p38 MAPK, as well as endogenous non coding single stranded small RNAs (miRNAs), regulate the expression of cell cycle related factors and proteins such as Cyclin D1/E, CDKs, c-Myc, and c-Src, thereby inducing cell cycle arrest at multiple stages, with the G0/G1 phase being the most common, thereby inhibiting tumor cell proliferation.)

## 3 Induction of apoptosis in tumor cells

Apoptosis is a programmed and proactive process of cell death that occurs under a series of complex and finely expressed genes (D’arcy, 2019). Apoptosis plays an important role in maintaining body homeostasis and anti-tumor, and inducing tumor cell apoptosis is the key mechanism of Naringin’s anti-tumor effect.

### 3.1 Mitochondrial pathway and death receptor pathway

Ramesh (Ramesh and Alshatwi, 2013) and Banjerdpongchai (Banjerdpongchai et al., 2016) et al. found that Naringin can induce apoptosis of cervical cancer SiHa cells and liver cancer HepG2 cells, and its mechanism is to upregulate the expression of endogenous promoter caspase-3 and -9, pro apoptotic protein Bax, P53, downregulate the expression of anti apoptotic protein Bcl xL, and induce apoptosis through the mitochondrial pathway; Upregulate the expression of Transmembrane protein Fas, its adaptor protein FADD, and endogenous promoter caspase-8, and induce cell apoptosis through death receptor pathway. Naringin can also inhibit the expression of mTOR in gastric cancer AGS cells (Raha et al., 2020), upregulate the expression of Bad, and downregulate the expression of Bcl xL, resulting in mitochondrial dysfunction, and then promote AGS apoptosis. Zeng et al. (Zeng et al., 2014) confirmed that Naringin inhibits NF- κ Activation of B/COX-2-caspase-1 pathway and downregulation of NF- κ The expression of Bp65, COX-2 and Cysteine protease 1 (caspase-1) upregulates the expression of cleaved caspase-3, thus inducing death receptor mediated apoptosis of cervical cancer HeLa cells. Further research by Wenjing et al. (Wen-jing et al., 2016) suggests that inhibiting HeLa growth by downregulating the relative expression of cyclooxygenase-2 (COX-2) may be its basic mechanism of action.

### 3.2 Endoplasmic reticulum pathway

Albayrak, Weili and others found that Naringin can induce reactive oxygen species (ROS) mediated Endoplasmic reticulum stress and activate PERK/eIF2 α/The ATF4/CHOP axis upregulates Bax and downregulates the level of B-lymphomatoma-2 gene (Bcl-2), thereby promoting apoptosis in colon cancer HT29 cells (Albayrak et al., 2021) and thyroid cancer B-CPAP cells (Li et al., 2022). Apoptosis of cervical cancer CC cells (C33A, SiHa and HeLa) also depends on Endoplasmic reticulum stress (Lin et al., 2020). By increasing the expression levels of apoptosis related proteins CHOP, PARP1 and caspase-3, eIF2 α Phosphorylation, activation of eIF2 α.

### 3.3 Regulation of miRNAs

Micro RNAs (miRNAs) are a class of non coding single stranded RNA molecules that participate in the post transcriptional level of Regulator gene expression and have functions such as regulating tumor cells, proliferation and apoptosis (He et al., 2020). Zhang et al. (Zhang et al., 2017; Yong-hui et al., 2021) verified that Naringin can directly inhibit its target gene KIAA1199 by increasing the expression of miR-216a, thereby promoting apoptosis of CRC cells in colorectal cancer. Yang Ruiying et al. (Rui-ying et al., 2022) proposed that Naringin can also promote the expression of Bax protein, reduce the expression of Bcl-2 and inhibit the apoptosis of cervical cancer ME-180 cells by up regulating miR-628-5p.

### 3.4 Multiple signaling pathways involved in inducing tumor cell apoptosis

In esophageal cancer Eca109 cells (Feng et al., 2021), Naringin can upregulate the expression levels of BAX, Cytochrome c oxidase (CytC), caspase-3 and -9, downregulate the expression of Bcl2, inhibit the JAK/STAT signaling pathway, and promote the apoptosis of Eca109 cells. Several studies have reported that Naringin can upregulate the expression of Caspase-3, cleaved Caspase-3 and Bax in thyroid cancer TPC-1 and SW1736 (Zhou et al., 2019), glioma U87 (Wei et al., 2020), and gastric cancer SUN-1 cell (Xu et al., 2021), and downregulate the expression of survivin and Bcl-2, thereby inducing cell apoptosis, by inhibiting the activation of PI3K/AKT pathway. Naringin inhibits PTEN/Akt signaling pathway through (Zhu et al., 2020), reduces the expression levels of Bcl-2, Bcl xL and survivin proteins, and increases the levels of caspase-3 and caspase-7, thereby inducing apoptosis of ovarian cancer SKOV3 cells. In Melanoma A375 and A875 cells (Guo et al., 2016), Naringin inhibits the phosphorylation of non Receptor tyrosine kinase c-Src, downregulates the expression of Bcl2, upregulates the expression of caspase-3 and Bax, and exerts apoptosis inducing activity by blocking the c-Src/AKT signaling pathway; In lung cancer H1299 cells (Xuemei et al., 2018), Naringin also plays a role in promoting apoptosis by regulating AKT signaling pathway, reducing Bcl2, and increasing caspase-3 and Bax levels. In addition, Kuang Liqiong et al. (Liqiong and Na, 2020) believe that the mechanism of Naringin promoting apoptosis of colon cancer SW620 cells is related to the regulation of the tumor suppressor gene ARHI. Naringin increases the expression of Bax and reduces the expression of Bcl-2 by increasing the level of ARHI.

In summary, Naringin induced apoptosis in tumor cells mainly by regulating the Caspase family (Caspase-1, -3, -7, -8, -9, etc.) and Bcl-2 family (Bcl-2, Bcl-xL, Bax, Bad, etc.), and its mechanism of action was related to the regulation of 3 basic pathways (mitochondria, endoplasmic reticulum, death receptors), inhibition of JAK/STAT pathway, Akt related pathway, CHOP related pathway, regulation of microRNAs (miR-216a, miR-628-5p), and regulation of the expression of apoptosis-related factors and proteins (survivin, P53, Fas, COX-2, CytC, etc.).

## 4 Inhibition of tumor cell invasion and metastasis

Invasion and metastasis of tumor cells means that tumor cells leave the primary focus, break through the Basement membrane and infiltrate into the surrounding stroma along the tissue gap; With the continuous growth of the invasive tumor, metastasis begins to occur. The dropped cancer cells enter the lumen through capillaries/Lymphatic vessel and are transported to the far side with circulation, or are directly planted and disseminated, and continue to proliferate in the secondary area to form a metastatic focus. Inhibition of tumor invasion and metastasis is one of the important anti-cancer mechanisms of Naringin.

Epithelial mesenchymal transition (EMT) refers to the process of epithelial cells transforming into mesenchymal phenotype cells, which is considered the initial cause of tumor invasion and metastasis (Mittal, 2018). The cadherin family is mainly involved in stabilizing intercellular connections and is a key factor in EMT (Cao et al., 2019). EMT is also closely related to changes in miRNA (Pan et al., 2021) and microenvironment (Aggarwal et al., 2021).

Naringin can inhibit the invasion and migration of colorectal cancer cells HCT116 and LOVO by inhibiting miR-216a and regulating the expression of CEMIP protein below its direct target KIAA1199 (Zhang et al., 2017); It can promote the expression of E-cadherin and reduce N-cadherin by regulating the miR-628-5p pathway, thereby blocking EMT and inhibiting the invasion and metastasis of cervical cancer ME-180 cells (Rui-ying et al., 2022). In addition, in thyroid cancer B-CPAP cells, Naringin regulates PERK/eIF2 α/ATF4/CHOP axis can upregulate the expression of E-cadherin and downregulate the expression of N-cadherin and Vimentin, which can significantly inhibit tumor cell metastasis and invasion (Li et al., 2022). According to Erdogan’s report (Erdogan et al., 2018), Naringin also inhibits the expression of zinc finger protein transcription factor Snail and basic helix loop helix transcription factor Twist by inhibiting NF kB/ERK signaling pathway, hindering the EMT process, so as to weaken the invasion and migration of prostate cancer DU145 and PC3 cells.

The change of microenvironment has a significant impact on EMT. The degradation of extracellular matrix and Basement membrane, which constitute the microenvironment, is a key link in tumor invasion and metastasis (Taki et al., 2021). Matrix metalloproteinase (MMPs) are enzymes responsible for degrading extracellular matrix, of which MMP2 and MMP9 play a prominent role (Abdel-Hamid and Abass, 2021). The research of Guo and Wang Wei et al. shows that Naringin can significantly reduce the expression of its downstream signal MMP2 and MMP9, and inhibit the migration and invasion potential of Melanoma A375 cells and glioma U87 by inhibiting the phosphorylation of c-Src (Guo et al., 2016) and inhibiting the PI3K/AKT pathway (Wei et al., 2020), respectively. In addition, Aroui et al. (Aroui et al., 2016) observed that in addition to the decreased expression and activity of MMP-2 and MMP-9, the expression level of TIMP-1/2 was significantly increased in Glioblastoma U251 cells, which was related to the inactivation of p38 signaling pathway by Naringin. Angiogenesis is the source of oxygen and nutrition needed for tumor survival and an important channel for tumor proliferation and metastasis. As one of the most important angiogenic factors, VEGF can promote the expression of p-VEGFR2 protein, induce the degradation of Basement membrane (Shah et al., 2021), and produce some key enzymes and proteins such as MMPs, MIC, uPA and TTPA. These enzymes and proteins can promote the migration and invasion of endothelial cells (Hua-ying and Debing, 2012). In addition, in his research on Glioblastoma U-87 cells (Aroui et al., 2020), he also found that Naringin inhibits VEGF induced VEGFR2 production by blocking ERK-P38-JNK signaling pathway, and downregulates the expression of endothelial cell markers CD31 and CD105 mRNA, suggesting that Naringin has a certain impedance effect on glioma angiogenesis.

In summary, Naringin mainly inhibits the invasion and metastasis of tumor cells by regulating invasion and migration-related factors and proteins such as MMP-2 and MMP-9, Snail/Twist, N-cadherin/E-cadherin, and Vimentin, and thus regulates related miRNAs, thereby inhibiting the EMT process. Its mechanism is related to the inhibition of signaling pathways such as c-Src, NF-kB/Akt/ERK, and ERK-P38-JNK.

## 5 Induction of autophagy in tumor cells

Cellular autophagy is a molecular regulation related to autophagy that relies on degradation mechanisms and uses lysosomes to remove their own damaged, degenerated, or senescent components in order to maintain normal cell growth and homeostasis (Hong and Rong-guang, 2016). Autophagy reduces tumorigenicity based on waste removal, structural reconstruction, immune protection, and suppression of cellular stress responses (Maiuri et al., 2007). Induction of autophagy in tumor cells is one of the mechanisms by which Naringin exerts its antitumor effects.

The autophagy-related proteins LC3 and p62 (Jiang and Mizushima, 2015), and the yeast Atg6 homolog (Beclin-1) (Xu and Qin, 2019), a key molecule in autophagy, are key factors in the autophagic process. Beclin-1 is involved in the formation of autophagosomal membranes and can effectively activate the initiation of autophagy; p62 plays an important regulatory role in the autophagic process and can perform autophagic degradation by binding to ubiquitinated proteins; and LC3 is the key to determining whether autophagy is activated. Bad et al. BH3-only pro-apoptotic proteins can release Beclin-1 by competitively binding to the anti-apoptotic factor Bcl-2/Bcl-xL, thus inducing autophagy in cells (D’arcy, 2019). MTOR is considered to be the most typical negative regulator of autophagy, PI3K/Akt/mTOR, and is usually activated in cancer cells (Wang and Zhang, 2019).

Raha et al. (2015). observed cytoplasmic vesicle and autophagosome formation in naringenin-treated AGS cells and found that naringenin-induced autophagy in gastric cancer AGS cells by activating the MAPK pathway, downregulating the cascade, and upregulating the autophagy proteins Beclin-1 and LC3B and phosphorylated mitogen-activated protein kinases (MAPKs). In addition, he also found in another study (Raha et al., 2020) that Naringin activates the p38/MAPKs pathway mediated by reactive oxygen species (ROS), and releases the lysosomal protein hydrolase Cathepsin D, upregulates Bad, ERK1/2 protein expression, which in turn triggers lysosomal membrane permeability (LMP)-mediated lysosomal cell death and induces autophagy. In gastric cancer SNU-1 cells, Naringin similarly activated autophagy in gastric cancer SNU-1 cells by inhibiting the PI3K/AKT signaling pathway, upregulating the expression of LC3B and Beclin-1 and downregulating the expression of p62 (Xu et al., 2021).

In summary, Naringin contributes to autophagy factor-dependent cell death and LMP-mediated lysosome-dependent cell death mainly through upregulation of autophagy-related proteins Beclin 1, LC3B, and Bad, and downregulation of p62 and mTOR protein expression, and its mechanism of action is related to the inhibition of PI3K/AKT and p38/MAPKs pathways.

## 6 Reverse tumor cell drug resistance and enhance chemotherapy drug sensitivity

Currently, chemotherapy is still the main method for treating malignant tumors, but because cancer cells are prone to develop resistance to chemotherapeutic drugs, it often leads to reduced efficacy or even treatment failure (Nussinov et al., 2021). Reversing drug resistance of tumor cells and improving their sensitivity to chemotherapeutic drugs is one of the key mechanisms by which Naringin exerts its anticancer effects.

NF-κB activity is significantly negatively correlated with drug sensitivity, and the NF-κB signaling pathway is an important pathway mediating drug resistance in tumor clusters (Mortezaee et al., 2019). In addition, the ABC transporter protein family is closely associated with multi-drug resistance (MDR), the most representative of which is the multidrug resistance protein P-glycoprotein (P-gp), multidrug resistance-associated protein (MRP), whose overexpression or increased activity can lead to the development of MDR (Ya-qiong et al., 2016). According to Zhu et al. (2020), Naringin partially reversed the expression of platinum drugs such as cisplatin in ovarian cancer SKOV3/CDDP cells by down-regulating the expression of P-gp protein, epoxygenase-2 (COX-2), down-regulating the expression of drug resistance genes MDR1 mRNA, MRP2 mRNA, and MRP2 protein, reducing intra-tumor cellular chemotherapeutic drug efflux, and increasing drug concentration The reversal mechanism was related to the blockade of NF-κB signaling pathway. A study by Mei-ying et al., 2019 showed that Naringin increased the uptake of cisplatin and decreased its efflux and sensitivity to cisplatin in lung-cancer resistant strain A549/DDP cells by upregulating Bax expression and downregulating P-gp, MRP1, p-Akt, and chemokine receptor CXCR4 protein levels. Erdogan (Erdogan et al., 2018) found that the use of Naringin and paclitaxel combination therapy significantly enhanced the cytotoxic effect of paclitaxel on prostate cancer (PC) DU145 and PC3 cells and LNCaP cells. All of these suggest that Naringin can enhance the sensitivity of tumor cells to platinum drugs and paclitaxel, and has good sensitizing properties.

In conclusion, Naringin mainly inhibits the signaling pathways such as Akt/NF-κB by down-regulating the expression of drug resistance gene MDR mRNA and related proteins P-gp and MRP, thus reversing the resistance of tumor cells to chemotherapeutic drugs, and its combination with chemotherapeutic drugs can significantly enhance the sensitivity of cancer cells to the drugs.

## 7 Others

### 7.1 Anti inflammation can prevent the occurrence of cancer

A variety of cytokines are involved in the inflammatory response, and the persistent inflammatory stimuli can damage the body tissues and disrupt the homeostasis in the body, and participate in the construction of the tumor microenvironment by altering the homeostasis in the tumor tissues, which has a significant pro-cancer effect (Denk and Greten, 2022). Therefore, anti-inflammation is also one of the mechanisms of action by which Naringin exerts its anticancer pharmacological activity.

Under the stimulation of chronic inflammation and intracellular infection, NF-κB, the primary response to oxidative stress, is activated and cyclooxygenase-2 (COX-2) containing its binding site is increased (Jia-jie and Bei-cheng, 2014). In a male C57BL/6 mouse model (Zhang et al., 2016), Naringin decreased interleukin-6 (IL-6) secretion by inhibiting the NF-κB/IL-6/STAT3 pathway, downregulated the expression of COX-2, myeloid-derived suppressor cells (MDSCs), proinflammatory mediators GM-CSF/M-CSF, and tumor necrosis factor-α (TNF-α), and increased the number of macrophages, CD4^+^ T and CD8^+^ T cells thereby slowing down the growth of colitis and colorectal adenoma cells and reducing the possibility of carcinogenesis. In another study on adenomatous colorectal polyposis (ApcMin/+) male mice (Zhang et al., 2018), Zhang et al. found that Naringin also inhibited the activity of the above pro-inflammatory mediators and factors through inhibition of GSK-3β/APC/β-catenin pathway for the prevention and treatment of intestinal tumors.

### 7.2 Mitigation of chemotherapeutic drug adverse reactions

The use of chemotherapeutic agents is usually inevitably accompanied by systemic or local adverse reactions (Zraik and Hess-Busch, 2021). Liu et al., 2017 showed that the combination of adriamycin and Naringin was more effective in inhibiting the growth of cervical cancer HeLa cells and promoting apoptosis of HeLa cells *in vitro* and *in vivo* (5–6 week-old female thymus-free nude mice). Compared with the adriamycin regimen alone, the combination of Naringin resulted in a significant reduction in anthracycline antitumor drugs The rapid weight loss and cardiac and hepatic, and renal toxicity caused by Naringin were significantly reduced. Elsawy H et al. (Elsawy et al., 2020) showed that Naringin can significantly reduce methotrexate induced serum alanine Transaminase (ALT), Aspartic acid Transaminase (AST), Alkaline phosphatase (ALP) and total bilirubin (TBIL) levels, reduce methotrexate induced liver Interleukin 6 (IL-6) and tumor necrosis factor-a (TNF-a) production, and also reduce liver Malondialdehyde (MDA) and nitric oxide (NO) content, Increase the contents of Superoxide dismutase (SOD), Catalase (CAT), Glutathione peroxidase (GPx), Glutathione reductase (GR) and glutathione (GSH) to alleviate the oxidative stress induced by methotrexate. Gelen et al., 2018 found that compared with the Naringenin+5-fluorouracil (5-FU) group, the serum creatinine (Cr) and Blood urea nitrogen (BUN) of the rats treated with 5-fu alone were significantly increased, and the hepatocytes in the surrounding area of the center were necrotic. It is suggested that Naringin can improve the liver and kidney injury induced by chemotherapy drugs, and has a protective effect on liver and kidney.

### 7.3 Enhancement and activation of immunity

The immune system has the functions of supervision, defense and regulation. It can detect the specific antigen expressed by tumor cells, recognize and kill tumor cells by activating and activating specific immune cells, and effectively inhibit the growth and spread of tumors (Wu et al., 2021). In addition, the immune system can further mediate the implementation of anti-tumor therapy, thereby improving anti-tumor efficacy. A weak immune system can reduce resistance to pathogens.

The induction of immune response depends on the activity of Antigen-presenting cell (APCs), such as a large number of macrophages in the spleen, lymph nodes and lymph sinuses. It has been proved that macrophages under the CD169 positive envelope preferentially participate in antigen presentation and anti-tumor immune response (Gasteiger et al., 2016). Fujiwara et al. measured the effect of 50 natural compounds on the expression of CD169 in macrophages, and found that five compounds enhanced the expression of CD169, especially Naringin. They conducted experiments *in vitro* and *in vivo*, and found that Naringin can induce lymph node macrophages to activate into CD169 positive phenotype, fully upregulate the expression of CD169 in macrophages, thereby enhancing anti-tumor immunity (Fujiwara et al., 2018). Another study showed that after taking citrus juice containing Naringin and Naringenin for 2 weeks, lymphocytes in the human body proliferated significantly, and the activity of Natural killer cell was significantly improved (Miles and Calder, 2021). The above results suggest that Naringin has an obvious enhancement and activation effect on the immune system.

The antitumor effects and mechanism of action of Naringin are summarized below. See Table 1.

**TABLE 1:** Anti-tumor action and mechanism of action of Naringin.

Pharmacological action	Cell/Animal model	Mechanism of action
Blocking the tumorcell cycle and Inhibiting the tumorcell proliferation	Cervical Cancer C33A, HeLa (Lin et al., 2020), Stomach Cancer SNU-1 (Xu et al., 2021)	Inhibit PI3K/AKT, β-catenin/GSK-3β pathway; ↓c-Myc, Cyclin D1, CDK2, CDK4; ↑p21/cip1, p27/kip1; G0/G1 phase block
	Prostate Cancer PCa (Erdogan et al., 2018)	Inhibit NF-kB pathway; ↓p50, ↑p21, p27, p53; G0/G1 phase block
	Triple-negative breast cancer MDAMB-231, BT-549 (Li et al., 2013)	Regulate the Wnt/β -catenin pathway; ↑p21, ↓p-Rb, Cyclin D1, Cyclin E; G0/G1 phase block
	Breast Cancer (El-Kersh et al., 2021)	Antiestrogenic and Inhibit-aromatase activity
	Colon and Rectal SW620 (Cheng et al., 2020a; Liqiong and Na, 2020)	Inhibit PI3K/AKT/mTOR pathway; ↓p-mTOR, CyclinD1, ↑ARHI, p21
	Osteosarcoma MG63, U2OS (Ming et al., 2018)	↓Zeb1, ↓Cyclin D1; G1 phase block
	Bladder Cancer 5637 (Kim et al., 2008)	Inhibit Ras/Raf/ERK pathway, ↑p21WAF1/cip1 ↓cyclin D1/E, CDK2, CDK4; G1 phase block
	Osteosarcoma MG63, U2OS (Yi et al., 2018)	↓Zeb1, ↓Cyclin D1; G1 phase block
	Ovarian Cancer SKOV3/CDDP (Zhu et al., 2020)	Inhibit Wnt/catenin pathway; ↓β-catenin, c-Myc, cyclin D1
		G1/S phase block
	Cervical Cancer SiHA (Ramesh and Alshatwi, 2013)	G2/M phase block
	Colon and Rectal HCT116 (Cheng et al., 2020a), Stomach Cancer AGS (Raha et al., 2020)	Inhibit PI3K/AKT/mTOR pathway, ↓p-mTOR
	Triple-negative breast cancer (Raha et al., 2020)	Regulate Wnt/β-catenin pathway
	NSCLC Lung CancerA549, H460 (Zhongyuan et al., 2017)	Inhibit PI3K/AKT/mTOR pathway, ↓p-mTOR,↓p70S6K
	Melanoma A375 (Bing-yu et al., 2016)	Inhibit c-Src/AKT pathway; ↓PKM2, LDHA, HIF-1α; Inhibit aerobic glycolysis
	Glioblastoma U87 (Wei et al., 2020)	↓CDK4/CDK6
	Cervical Cancer ME-180 (Rui-ying et al., 2022)	↑miR-628–5p
	Non-small cell lung cancer H69 (Chen et al., 2018)	Regulate NF-κB pathway, ↓VCAM-1, ↑ miR-126
	Melanoma A875 (Guo et al., 2016)	Inhibit c-Src/AKT pathway; ↓CDK4/CDK6, ↓PKM2, LDHA, HIF-1α; Inhibit aerobic glycolysis
	Esophageal cancer Eca109 (Feng et al., 2021)	Inhibit the JAK/STAT pathway,↓p-STAT3/STAT3, ↓p-JAK2/JAK2
Inducing tumor cell apoptosis	Stomach Cancer AGS (Raha et al., 2020)	The mitochondrial pathway; ↓mTOR, Bcl-xL; ↑Bad
	Cervical Cancer ME-180 (Rui-ying et al., 2022)	↑miR-628–5p, Bax; ↓Bcl-2
	Thyroid Cancer TPC-1, SW1736 (Zhou et al., 2019); glioma U87 (Wei et al., 2020), Stomach Cancer SUN-1 (Xu et al., 2021)	Inhibit PI3K/AKT pathway, ↑Caspase-3, cleaved Caspase-3, Bax; ↓survivin, Bcl-2
	Melanoma A375, A875 (Guo et al., 2016)	Inhibit c-Src/AKT pathway, ↓Bcl2, ↑caspase-3, Bax
	Esophageal cancer Eca109 (Feng et al., 2021)	↑BAX, CytC, caspase-3, -9, ↓Bcl2, Inhibit JAK/STAT pathway
	Cervical Cancer SiHa (Ramesh and Alshatwi, 2013), Liver Cancer HepG2 (Banjerdpongchai et al., 2016)	The mitochondrial pathway; ↑caspase-3, caspase-9, Bax, P53↓Bcl-xL; The death receptor pathway; ↑Fas, FADD, caspase-8
	Cervical Cancer HeLa (Zeng et al., 2014)	↓NF-κB p65, COX-2, caspase-1; ↑cleaved caspase-3
	Colon Cancer HT29 (Albayrak et al., 2021), Thyroid Cancer B-CPAP (Li et al., 2022)	ROS-mediated ER stress; activate PERK/eIF2α/ATF4/CHOP pathway, ↑Bax, ↓Bcl-2
	Cervical Cancer C33A (Lin et al., 2020)	Endoplasmic reticulum pathway; ↑CHOP, PARP1, caspase-3, activate eIF2α, ↑miR-628–5p, Bax, ↓Bcl-2
	Ovarian Cancer SKOV3 (Zhu et al., 2020), Lung Cancer H1299 (Xuemei et al., 2018)	Inhibit PTEN/Akt pathway, ↓Bcl-2, Bcl-xL, survivin; ↑caspase-3, caspase-7
	Colon Cancer SW620 (Liqiong and Na, 2020)	Regulate AKT pathway, ↓Bcl2, ↑caspase-3, Bax; ↑ARHI, Bax; ↓Bcl-2
Inhibit invasion and metastasis	Colon and Rectal HCT116, LOVO (Albayrak et al., 2021)	Inhibit miR-216a, ↓KIAA1199,↓CEMIP
	Thyroid Cancer B-CPAP (Li et al., 2022)	Regulate PERK/eIF2α/ATF4/CHOP, ↑E-cadherin, ↓N-cadherin, Vimentin
	Melanoma A375 (Guo et al., 2016)	Inhibit c-Src/PI3K/AKT pathway, ↓MMP2, MMP9
	Cervical Cancer ME-180 (Rui-ying et al., 2022)	Regulate miR-628–5p pathway, ↑E-cadherin, ↓N-cadherin, hinder EMT
	Prostate Cancer DU145, PC3 (Erdogan et al., 2018)	Inhibit NF-kB/ERK pathway, ↓Snail, Twist, hinder EMT
	Glioblastoma U251 (Aroui et al., 2016)	Inactivate p38 pathway, ↓MMP-2, MMP-9, ↑TIMP-1/2
	Glioblastoma U87 (Aroui et al., 2020)	Inhibit ERK/P38/JNK pathway, ↓VEGFR2, ↓CD31, CD105mRNA, anti-angiogenic
Induce autophagy of tumor cells	Stomach Cancer AGS (Raha et al., 2015)	Activate MAPK pathway, Inhibit PI3K/Akt/mTOR pathway, ↑Beclin-1, LC3B, MAPKs
	Stomach Cancer SNU-1 (Xu et al., 2021)	Activate ERK/p38 pathway,↑Cathepsin D, ↑BH3-only Bad, ERK1/2, induce LMP mediated lysosomal cell death; Inhibit PI3K/AKT pathway, ↑LC3B, Beclin-1, ↓p62
Reverse tumor cell drug resistance and increase sensitization	Ovarian cancer SKOV3/CDDP (Zhu et al., 2020)	Inhibit NF-κB pathway, ↓P-gp, COX-2 ↓MDR1 mRNA, MRP2 mRNA, MRP2
	Prostate Cancer DU145, PC3, LNCaP (Erdogan et al., 2018)	Enhance the cytotoxic effects of paclitaxel
	Lung Cancer persister A549/DDP (Mei-ying et al., 2019)	↑Bax, ↓P-gp, MRP1, p-Akt, CXCR4
Anti-inflammatory	C57BL/6 Male mousemodel (Zhang et al., 2016)	Inhibit NF-κB/IL-6/STAT3 pathway, ↓COX-2, MDSCs, GM-CSF/M-CSF, TNF-α; ↑ macrophage, CD4+T, And CD8 + T cell number
	(ApcMin/+)Male mouse model (Zhang et al., 2018)	Inhibit GSK-3β/APC/β-catenin pathway, ↓Cox-2, TNF-α, PGE2; ↓IL-6
Reduce adverse reactions	Cervical Cancer HeLa (Liu et al., 2017)	Improve weight loss, reduce cardiotoxicity, hepatorenal toxicity ↓ALT、AST、ALP、TBIL; ↓IL-6、TNF-a; ↓MDA、NO; ↑ SOD、CAT、GPx、GR、GSH; ↓Cr、BUN
	Liver tissue of Male albino rats (Elsawy et al., 2020)	
	Liver and kidney tissues of male adult Sprague-Dawley rats (Gelen et al., 2018)	
Enhance and activate immunity	Human monocyte-derivedmacrophages, The C57BL/6 n mouse (Fujiwara et al., 2018)	Activate lymph node CD169 positive macrophages, ↑ CD169

## 8 Discussion and outlook

(Natural compounds are chemical substances that exist in nature and are produced by organisms such as animals, plants, and microorganisms. There are many types and quantities of natural compounds, which usually have rich structural diversity, biological activity, and pharmacological activity. In addition, compared to other synthetic drugs, natural compounds have a shorter average half-life and higher biodegradability; It does not directly compete with synthetic compounds and can usually be used as a supplement or reference for synthetic drugs or chemicals; It is more biocompatible to the human body and has fewer serious side effects. At present, many Chinese herbal monomer compounds have been used in the research, development and treatment of antibiotics, anti-cancer drugs, cardiovascular drugs, etc., such as Flavonoid, terpenes, polysaccharides, alkaloids, etc. (Wang et al., 2021). Researchers are constantly looking for new anti-tumor drug candidates or chemicals from them, and expanding the field of anti-tumor Drug development and chemical research (Wen et al., 2021). Naringin is one of the natural dihydro Flavonoid (Alam et al., 2022), its Molecular formula is C27H32O14, its relative molecular weight is 580.54 g/mol, chemically 4′, 5,7-trihydroxyflavanone-7-rhamnoglucoside; Naringenin is the aglycone of Naringin (Motallebi et al., 2022), which forms a glycoside with Neohesperidose at position 7. In many studies, Naringin and Naringenin have been proved to have multiple effects such as anti-tumor (Rauf et al., 2022), anti-inflammatory and antiviral (Tutunchi et al., 2020), antioxidant, hypoglycemic (Blahova et al., 2021), cholesterol (Wang et al., 2020), gastrointestinal protection (Cao et al., 2018), prevention and treatment of bone injury (Ge and Zhou, 2021), neuroprotection (Emran et al., 2022), antidepressant (Hernández-Vázquez et al., 2022), cardio cerebral protection (Moghaddam et al., 2020), and are effective, safe, and well tolerated biological Flavonoid.

### 8.1 Advantages and characteristics of naringin

Compared with other flavonoids, Naringin has obvious advantages. First of all, as mentioned above, Naringin and its aglycone have very extensive and comprehensive effects, which is a treasure house with great potential worth tapping; Secondly, from the perspective of pharmacokinetics, some widely studied traditional Chinese medicine monomers with anti-tumor activity, such as Curcumin (Feng et al., 2017a), Baicalin (Huang et al., 2019), Quercetin (Alizadeh and Ebrahimzadeh, 2022), have poor water solubility, low absorption rate, low bioavailability, fast metabolism and systemic clearance, and their effectiveness is limited. After Naringin is taken orally into the intestinal tract, it will basically undergo deglycosylation reaction and metabolize to Naringenin under the effect of Gut microbiota, It is rapidly absorbed into the bloodstream through the intestinal wall and can be detected in plasma within 5 min. Its presence can be detected in all organs except for brain tissue, and it is widely distributed in the body (Pei-bo et al., 2020), indicating that it can efficiently exert therapeutic effects throughout the body. Naringin has hepatointestinal circulation, which not only improves the utilization rate, but also prolongs the duration of its action. In addition, Naringin has its own uniqueness (Yang et al., 2022), for example, Apigenin (Singh et al., 2022) and Curcumin (Stanić, 2017) basically regulate the production of cytokines at the transcriptional level, while Naringin not only inhibits the expression of cytokines mRNA, but also promotes the degradation of Lysosome dependent cytokines, providing a new idea for anti inflammation and strengthening immune regulation. Naringin can enhance the absorption of colchicine (Dahan and Amidon, 2009), strengthen the lipid-lowering effect of Atorvastatin (Sama et al., 2019), increase the uptake of cisplatin (Mei-ying et al., 2019) and paclitaxel by cancer cells (Choi and Shin, 2005), and reduce the efflux of anti-tumor drugs by regulating P-gp; It is suggested that Naringin can improve the biological activity of drugs and can be used as a drug enhancer. It is reported that the effect of Naringin on drug accumulation after high-fat diet and repeated administration is not significant (Bai et al., 2020); In acute, chronic and subchronic toxicity studies, no incidence rate, mortality and toxicology related events were observed during the administration period or in the recovery period after administration (Li et al., 2020); At present, no research has proved that pure Naringin, Naringenin or their food sources have adverse effects on human body (Pei-bo et al., 2020). All these show that Naringin is well tolerated, safe and reliable.

### 8.2 Development prospect of naringin in anti-tumor field

In combination with the characteristics and advantages of Naringin, Naringin has a broad development prospect in the field of anti-tumor and has great potential.

#### 8.2.1 Anti tumor potential

Naringin has been shown in laboratory and preclinical studies to have a clear inhibitory effect on a variety of tumor cells and a broad spectrum of anti-cancer effects. The anti-tumor mechanism of Naringin is closely related to multiple signal pathways and targets. They are involved in the whole process of tumor cell growth and death in multiple forms, multiple links, and all directions. They precisely regulate cell cycle, proliferation and apoptosis, invasion and migration, autophagy, inflammation related factors and proteins, and play an anti-tumor role together.

#### 8.2.2 Potential of comprehensive treatment

Naringin can be combined with other anti-tumor drugs or treatment methods to enhance the therapeutic effect, strengthen the sensitivity to tumor cells, and reduce the occurrence of drug resistance.

#### 8.2.3 Potential to reduce side effects

Compared with some commonly used anti-tumor drugs, Naringin has lower toxic and side effects. It originates from natural plants, has low toxicity to the human body, and has shown good tolerance and safety in some clinical practices.

### 8.3 Future issues to be solved and areas for improvement

Under the background of continuous social and medical progress, the coordinated, multi accurate comprehensive treatment mode has become the development trend of tumor treatment, including immunotherapy, Targeted therapy and other treatment methods, which play a vital role (Colli et al., 2017). However, there are few studies on Naringin combination therapy at present, and they focus on the combination with traditional chemotherapy drugs such as platinum, lacking basic and clinical research on Naringin combined with immunity, targeted drugs and other drugs for the treatment of malignant tumors. In addition to sensitizing cisplatin, paclitaxel and attenuated doxorubicin (Erdogan et al., 2018; Zhu et al., 2020), can Naringin achieve similar enhancement effect on other chemotherapy drugs? Can Naringin in combination with immunocheckpoint inhibitors, bioimmunotherapy or targeted drugs such as anti angiogenesis agents also achieve benefits? Further exploration is needed on the specific pharmacological mechanisms, toxic side effects, safety evaluation, optimal benefit plans, and scope of application of combined therapy. In addition, studies at home and abroad have confirmed that Naringin has a clear inhibitory effect on a variety of tumor cells and has a broad spectrum of anti-cancer effects. Is Naringin the best choice for a tumor or a tumor cell line? These new questions need to be answered and resolved in future research.

At present, most studies on Naringin are based on *in vitro* cell experiments and animal experiments, while there are few *in vivo* studies on human subjects. The complete metabolic pathway, process and mechanism of Naringin *in vivo* still need to be more clearly described. It is necessary to continue to explore the individual differences of Naringin pharmacokinetics, observe and improve its long-term drug safety, further carry out clinical research on human body, verify the safety and effectiveness of Naringin in human body, provide data support for *in vivo* and clinical research for individualized drug delivery, and help guide clinical more scientifically. The use of Naringin alone as an anti-tumor drug may have limited effect. Due to factors such as Naringin metabolism *in vivo* and Drug interaction, its specific application mode and dosage need further research and optimization.

In addition, Naringin has low water solubility (Rivoira et al., 2021) and oral Malabsorption. In order to make its related products widely developed, we need to constantly improve its bioavailability. The following aspects can be considered:

#### 8.3.1 Improve solubility

Increase its dissolution rate and drug absorption in the gastrointestinal tract through technical means, such as nano treatment, solid dispersion, Inclusion compound, liposome, etc. (Zhong et al., 2014). At present, the application of Naringin in nano field is a research hotspot. Nanoparticle materials, water-soluble nanocapsules, nano lotion, etc. can be used as drug carriers, which can effectively encapsulate, deliver and slow release Naringin (Feng et al., 2017b). Nanocarriers can achieve targeted delivery of Naringin, improve its stability *in vivo*, drug concentration and retention rate, and reduce the toxic and side effects of drugs on non target cells (Cheng L. et al., 2020). After encapsulation, Naringin can still maintain its good antioxidant capacity. Nanotechnology can also help to concentrate the administration and release of Naringin at specific sites, and improve the accumulation and therapeutic effect of drugs in tumor regions. Other ingredients can also be used together to enhance the solubility of Naringin, for example, the solubility of Naringin Neohesperidin in water boiling is significantly higher than that of Naringin alone (Hua and June 2021).

#### 8.3.2 Improve oral absorption

After oral administration, Naringin needs to be absorbed into the blood circulation through the gastrointestinal tract. To improve the oral absorption of Naringin, some strategies can be taken, such as increasing its fat solubility, improving its intestinal permeability, blocking intestinal degrading enzymes (Murota et al., 2018), etc. In the future, the drug structure can be modified, the complex of Gut microbiota regulator and Naringin can be developed (Feng et al., 2019), the dosage form and usage can be changed, and its stability under various processing and storage conditions can be explored to improve the drug utilization value as much as possible.

#### 8.3.3 Improve drug metabolism and excretion

Prolong the residence time and drug concentration of Naringin in the body by regulating its metabolic enzyme activity, reducing its metabolism speed in the body, or increasing the liver reabsorption and other means.

The active ingredients of traditional Chinese medicine are complex, and single medicine often has many components with pharmacological activity, such as orange peel, orange peel, other flavonoid monomers contained in orange peel, citrinin, Naringin, hesperidin, and their volatile oils have been proved to have some significance in anti-tumor (Kun et al., 2015). Is Naringin, as one of the effective active ingredients, the most critical and effective anti-tumor component in these traditional Chinese medicines? Further research on pharmacological effects and molecular mechanisms is needed to verify in the future. The research on the mechanism of Naringin mostly stays at the overall level. In clinical practice, the combination of multiple medicines is often used in the form of compound medicine. At present, comprehensive studies on some compound medicines and multiple ingredients are common, so it is not easy to clarify the efficacy and value of Naringin alone. On the other hand, there are few existing studies on traditional Chinese medicine compounds, and there are few discussions on the anti-cancer mechanism of Naringin based on the syndrome differentiation and treatment system of traditional Chinese medicine, which cannot reflect the close combination with traditional Chinese medicine. Therefore, it is necessary to strengthen the analysis of the mechanism of Naringin’s action in the classic compound medicine, single medicine, traditional Chinese patent medicines and simple preparations and related preparations under the guidance of the theory of Traditional Chinese medicine.

Naringin is a drug with great anti-tumor potential. Its development, application, development and application have broad prospects. We hope to continue to conduct in-depth excavation and research on it in the future, providing some reference and help for anti-tumor treatment, so as to improve the quality of life of cancer patients and extend their lives).
